# Streptococcus dysgalactiae Subspecies dysgalactiae Infection Presenting With Septic Shock

**DOI:** 10.7759/cureus.12465

**Published:** 2021-01-03

**Authors:** Balamurugan Nathan, Vivekanandan Pillai, S. Manu Ayyan, Anuusha SS, K N J Prakash Raju

**Affiliations:** 1 Emergency Medicine, Jawaharlal Institute of Postgraduate Medical Education and Research, Puducherry, IND; 2 Internal Medicine, Jawaharlal Institute of Postgraduate Medical Education and Research, Puducherry, IND

**Keywords:** streptococcus dysgalactiae subspecies dysgalactiae, septic shock, cellulitis, myositis, rhabdomyolysis

## Abstract

*Streptococcus dysgalactiae* has two main subspecies: *Streptococcus dysgalactiae* subspecies *dysgalactiae* (SDSD) and *Streptococcus dysgalactiae* subspecies *equisimilis *(SDSE). Although there are various case reports of SDSE causing clinical infection in humans, very few reports of SDSD causing human infections have been reported in the literature. As of date, there are only five case reports of infection with SDSD and all five patients survived the infection. We report a case of a 40-year-old male who presented with features of right upper limb cellulitis and went into septic shock. This report is unique as it presents the first report of SDSD causing fulminant sepsis in humans. Elevation of total fraction of creatinine kinase was also seen in our patient which could be due to myositis or rhabdomyolysis.

## Introduction

*Streptococcus dysgalactiae* has two main subspecies: *Streptococcus dysgalactiae* subspecies *equisimilis *(SDSE) and *Streptococcus dysgalactiae* subspecies *dysgalactiae *(SDSD) [1]. The former was proposed as the name for human strains and they are believed to cause a variety of infections in humans similar to *Streptococcus pyogenes* [1]. The latter was the name proposed for the strains of animal origin [1] and they are an uncommon cause of infection in humans. There are very few reports of this organism causing clinical infection in humans [2-5] and there are no reports of fulminant infection caused by SDSD. Here we report a middle-aged male who presented with features of right upper limb cellulitis and had septic shock. Our patient also had an elevated total fraction of creatinine kinase which was possibly due to myositis or rhabdomyolysis since such high levels of creatinine kinase are unlikely to be due to cellulitis.

## Case presentation

A 40-year-old male who was working as a cook in a college hostel, with no known comorbidities presented to our Emergency Department with complaints of swelling of the right upper limb for two days. On arrival at our emergency department, the patient was conscious, oriented, and afebrile. Initial assessment revealed a patent airway, pulse rate of 120/min, blood pressure of 74/50 mmHg, respiratory rate of 30/min, and a saturation of 94% in room air. He had a Glasgow Coma Score of E4V5M6 with no focal neurological deficits. The capillary blood sugar was 113 mg/dl. Arterial blood gas analysis revealed severe high anion gap metabolic acidosis (the anion gap measured 21.7 mmol/L and the bicarbonate measured 18.5 mmol/L) with a lactate of 4.3 mmol/L. The qSOFA (quick Sequential Organ Failure Assessment) score was two, and the electrocardiogram revealed sinus tachycardia with left axis deviation. We administered the RUSH (rapid ultrasound in shock) protocol which is a part of point-of-care ultrasound (POCUS) procedures. It revealed a hyperdynamic left ventricle with no regional wall motion abnormality and an inferior vena cava diameter of less than two centimeters with more than 50% inspiratory collapse. No vegetation was noted on the valves. The patient was initiated on a one-hour sepsis bundle. Two liters of Ringer’s lactate solution was infused initially as a part of fluid resuscitation. Blood cultures were obtained and he was empirically started on broad-spectrum antibiotics. Subsequently, he also required high dose double inotropic support (noradrenaline and vasopressin). 

A week prior to the presentation to our emergency department, the patient had developed a sore throat. Two days later he developed backache with generalized myalgia and was treated with analgesics for the same. Over the next two days, he also developed painful swelling of the right upper limb. There was no history of fever or trauma. Systemic examination revealed bilateral basal end-inspiratory fine crepitations. Local examination revealed only swelling of the right upper limb with no erythema, blisters, warmth, tenderness, or crepitus over the swelling. 

Laboratory investigations revealed elevated urea and creatinine, thrombocytopenia, hyperbilirubinemia, hypoalbuminemia, elevated aspartate aminotransferase (AST) and alanine aminotransferase (ALT), and elevated total fraction of creatine kinase (CK) (Tables 1, 2). The clinical and laboratory picture resembled septic shock with multiorgan dysfunction. Ultrasound examination of the right upper limb revealed subcutaneous thickening and interstitial edema suggestive of cellulitis. 

**Table 1:** Biochemical parameters of the patient

Laboratory parameter	Value	Reference range
Random blood glucose	144 mg/dL	70-140 mg/dL
Serum urea	99 mg/dL	15-40 mg/dL
Serum creatinine	2.3 mg/dL	0.7-1.2 mg/dL
Serum sodium	130 mEq/L	135-145 mEq/L
Serum potassium	3.3 mEq/L	3.5-5.0 mEq/L
Serum chloride	96 mEq/L	96-106 mEq/L
Serum calcium	7.4 mg/dL	8.8-10.6 mg/dL
Serum magnesium	2.1 mg/dL	1.8-2.6 mg/dL
Serum phosphorus	6.2 mg/dL	2.5-5.0 mg/dL
Serum uric acid	6.9 mg/dL	3.5-7.2 mg/dL
Total bilirubin	7.98 mg/dL	0.3-1.2 mg/dL
Direct bilirubin	5.27 mg/dL	0.0-0.20 mg/dL
Indirect bilirubin	2.71 mg/dL	0.3-1.2 mg/dl
Total protein	4.6 gm/dL	6.6-8.3 gm/dL
Serum albumin	2.5 gm/dL	3.5-5.2 gm/dL
AST (aspartate aminotransferase)	174 IU/L	0-50 IU/L
ALT (alanine aminotransferase)	70 IU/L	0-50 IU/L
Alkaline phosphatase	96 IU/L	30-120 IU/L
Gamma-glutamyltransferase	66 IU/L	0-55 IU/L
Lactate dehyrogenase	510 IU/L	208-378 IU/L
Serum amylase	113 IU/L	22-80 IU/L
Creatine kinase (total)	1384 IU/L	0-171 IU/L
Creatine kinase (MB)	71 IU/L	0-24 IU/L

**Table 2:** Hematological parameters of the patient

Hematological parameter	Value	Reference range
Hemoglobin	14.9 g/dL	13.5-16.5 g/dL
Red blood cell count	4.58 × 10^6^/ µL	4.7-6.1 x 10^6^/ µL
Hematocrit	43.2 %	38-50%
Mean corpuscular volume	94.3 fL	80-100 fL
Mean corpuscular hemoglobin	32.5 pg	27-32 pg
Total leucocyte count	6.14× 10^3^/ µL	4.0-11.0× 10^3^/ µL
Neutrophil	86.5%	40-75%
Eosinophil	0.8%	1-6%
Basophil	0.2%	0.5 to 1%
Lymphocyte	10.7%	20-45%
Monocyte	1.8%	2-10%
Platelet count	100× 10^3^/ µL	150-450× 10^3^/ µL

Although the patient was started on broad-spectrum antibiotics, he had a fulminant course and required vasopressin support in addition to noradrenaline (both in high doses). He sustained cardiac arrest following which he was resuscitated and connected to mechanical ventilation. But he expired on the same day of presentation due to refractory septic shock. Blood culture report later revealed infection with SDSD sensitive to levofloxacin, benzylpenicillin, ceftriaxone, and erythromycin.

## Discussion

Infections due to SDSD are not commonly seen in humans and very few case reports of infections in humans are documented in the literature. 

In 1996, Vandamme et al. subdivided *Streptococcus dysgalactiae* into *Streptococcus* subspecies *equisimilis *(the human pathogen) and subspecies *dysgalactiae *(the animal pathogen) [1].

SDSE primarily presents as skin and soft-tissue infections including pyoderma, cellulitis, wound infections, abscesses, erysipelas, and necrotizing fasciitis [6]. Invasive infections comprise arthritis, osteomyelitis, pleuropulmonary infections, peritonitis, intra-abdominal and epidural abscesses, meningitis, endocarditis, puerperal septicemia, neonatal infections, necrotizing fasciitis, myositis, and streptococcal toxic-like syndrome [6].

SDSD, a Lancefield group C streptococcus is a major cause of bovine mastitis and has been regarded as an animal‐restricted pathogen. It can cause cross-infection from animals to humans especially in those who are immunocompromised and those working in the fishing or animal industry [2]. SDSD has also shown the ability to cause severe cellulitis and toxic shock syndrome in cattle, septicemia in fish, and dogs [3].

SDSD has been reported to cause prosthetic joint infection [2], infective endocarditis [3], upper limb cellulitis [4], and lower limb cellulitis [5]. The patient with infective endocarditis reported by Jordal et al. had a complicated course but he survived [3]. A case of neonatal meningitis due to co-infection with SDSD and Herpes simplex virus has also been reported [7].

In all the above cases, the patients survived. However, in our case, the patient had a fulminant presentation with septic shock and multiorgan dysfunction syndrome and expired the same day despite the management of septic shock. The characteristics of patients in the various case reports are summarized in Table 3.

.

**Table 3:** Summary of patient characteristics and treatment details of the reported cases of infections due to Streptococcus dysgalactiae subspecies dysgalactiae DMARDs: disease-modifying antirheumatic drugs

Author and year	Patient characteristics	Underlying condition	Trauma/ exposure to animals	Source of infection/ diagnosis	Treatment given	Outcome
Koh et al., 2009 [4]	Mastectomy patient	Breast cancer treated	Fresh seafood contact	Upper limb cellulitis	Not specified	Recovered
Park et al., 2012 [2]	61-year male	Rheumatoid arthritis on DMARDS	Nil	Prosthetic joint infection	Vancomycin and ceftriaxone	Recovered
Jordal et al., 2015 [3]	65-year male	Nil	Nil	Endocarditis	Initially meropenem and vancomycin	Recovered
Chennapragada et al., 2018 [5]	49-year female	Multiple comorbid conditions	Nil	Lower limb cellulitis	Initially vancomycin, cefepime later changed to ceftriaxone	Recovered
Im et al., 2019 [7]	22-day neonate	Nil	Nil	Meningitis	Ampicillin	Recovered
Balamurugan et al., (this study)	45-year male	Nil	Nil	Cellulitis	Piperacillin/tazobactam and vancomycin	Died

Although rhabdomyolysis is very rare in infections due to group C streptococci [8], the elevation of the total fraction of creatinine kinase could point to the possibility of rhabdomyolysis. The other possibility for elevated creatinine kinase would be the presence of myositis as such levels of creatinine kinase are unlikely in patients with cellulitis. Streptococcal necrotizing myositis (SNM) can also be confused with cellulitis or deep vein thrombosis. SNM caused by group A beta-hemolytic streptococci usually affects healthy middle-aged males [9]. The mortality rate in necrotizing myositis ranges from 70 to 100 percent. Our patient also could have had myositis. However, we do not have evidence of myositis by muscle biopsy as no autopsy was done.

Lancefield group C streptococci are usually treated with penicillin. SDSE develops resistance to quinolone and macrolide not to penicillin and cephalosporin. The same treatment modalities for SDSE can also be used in the management of SDSD [6]. In our case, SDSD was sensitive to levofloxacin, benzylpenicillin, ceftriaxone, and erythromycin and although he was started on piperacillin-tazobactam, he did not respond clinically.

## Conclusions

Although SDSD is predominantly considered to be an animal pathogen, this case report highlights the fact that SDSD can cause septic shock in humans. The presence of elevated creatine kinase levels in our patient also points to the possible presence of myositis or rhabdomyolysis. Clinicians should be aware of the fulminant presentation of SDSD.
